# Peptides as a Therapeutic Alternative Against Leishmaniasis: A Scoping Review

**DOI:** 10.1111/cbdd.70314

**Published:** 2026-05-18

**Authors:** Maria Eduarda da Veiga Oliveira, Ellen Dyminski Parente Ribeiro, Otávio Henrique Pradi Guenther, Fernanda Tomiotto‐Pellissier

**Affiliations:** ^1^ Department of Medical Pathology Federal University of Paraná Curitiba Paraná Brazil

**Keywords:** antimicrobial peptides, leishmaniasis, therapeutics

## Abstract

Leishmaniasis is a neglected tropical disease with high global health and socioeconomic burden, particularly in low‐income regions where current treatments remain limited by high toxicity, cost, and increasing resistance. Antimicrobial peptides (AMPs), small bioactive molecules with broad‐spectrum activity and low propensity for resistance, have emerged as promising therapeutic candidates. This scoping review aimed to map the existing literature on AMPs with anti‐leishmanial activity, updating and expanding upon previous reviews. Following the PRISMA‐ScR framework, we systematically searched for primary research articles assessing natural and synthetic peptides through in vitro or in vivo approaches. Seventy‐nine studies met eligibility criteria, evaluating 231 unique peptides against multiple *Leishmania* species. Most investigations were in vitro, particularly on *L. major* promastigotes and amastigotes. Synthetic and bioengineered peptides demonstrated superior efficacy and selectivity compared with natural peptides, in some cases achieving selectivity indices exceeding 6000. Mechanistic studies revealed that AMPs act primarily through parasite membrane disruption, but also by inducing apoptosis‐like processes and modulating host immune responses. Despite their promise, several challenges remain, including peptide instability in physiological conditions, proteolytic degradation, and limited in vivo validation. Advances in chemical modifications and nanocarrier‐based delivery systems show potential to overcome these barriers. In conclusion, AMPs represent a compelling avenue for leishmaniasis therapy, especially for cutaneous forms of the disease. However, further studies, particularly in vivo, are critical to translate preclinical findings into clinical application.

## Graphical abstract

This scoping review of 79 studies identified 231 antimicrobial peptides with anti‐*Leishmania* activity, including natural‐derived and synthetic peptides (inspired by natural sources or developed through rational design). Most were evaluated in vitro against promastigote and/or amastigote forms, with synthetic peptides showing a more promising effect.

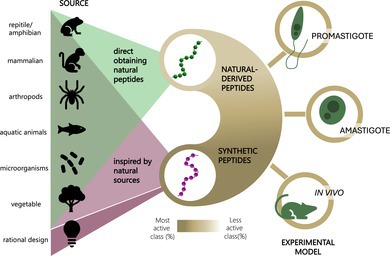

## 1 Introduction

Antimicrobial peptides (AMPs) serve as key molecules in the innate immune system of various organisms (N. Chen and Jiang 2023). Typically composed of 5–100 amino acids, these small, positively charged peptides exhibit broad‐spectrum antimicrobial activity against bacteria, protozoa, viruses, and other pathogens (C. H. Chen and Lu 2020).

Among the advantages of AMPs are their low toxicity to eukaryotic cells, strong thermal stability, high solubility, low molecular weight (T. Li et al. 2019), and versatility, as well as selectivity, tunability, and ease of synthesis (Robles‐Loaiza et al. 2022). These properties position them as promising candidates for medical applications, with several antimicrobial agents currently in clinical trials (Koo and Seo 2019).

Despite being a research focus for over 20 years, with more than 2000 AMPs identified from various organisms (Haney et al. 2011), the anti‐leishmanial activity of many AMP families has only recently been investigated (Robledo et al. 2023). Current research indicates that these peptides target Leishmania by disrupting the plasma membrane, increasing permeability, interacting with intracellular targets, and triggering apoptosis‐like biochemical changes (El‐Dirany et al. 2021). Additionally, within host tissues, AMPs appear to attract inflammatory cells to infection sites, indirectly inhibiting protozoa replication and viability (El‐Dirany et al. 2021).

Leishmaniasis is a vector‐borne disease prevalent in tropical and subtropical regions, caused by more than 20 species of *Leishmania*, which are trypanosomatid protozoa (Torres‐Guerrero et al. 2017). Leishmaniasis ranks as the second leading cause of death among protozoan diseases, after malaria, and is listed by the World Health Organization (WHO) as one of the seven most significant tropical diseases (Singh et al. 2023). Its effects are particularly catastrophic, as it predominantly affects impoverished and marginalized populations in underdeveloped regions, with significant prevalence in rural and peri‐urban areas (Valdivia et al. 2022).

The disease is classified into three main clinical syndromes: cutaneous leishmaniasis (CL), mucocutaneous leishmaniasis (MCL), and visceral leishmaniasis (VL) (Burza et al. 2018). VL, or kala‐azar, is the most severe form, causing systemic infections and has an 85%–90% fatality rate if untreated (Stockdale and Newton 2013). MCL affects mucous membranes and can result in significant facial disfigurement (Abadías‐Granado et al. 2021). CL, the most common form, presents as painless skin lesions that progress from papules to nodules with a central crust over a dry ulcer (Pace 2014).

The life cycle of Leishmania features two main forms: the extracellular, flagellated promastigote found in female sandflies (Phlebotomus or Lutzomyia genera) and the intracellular, non‐flagellated amastigote residing in the monocyte–macrophage cells of the mammalian host (Pace 2014). Amastigotes mature and proliferate within these phagocytes until the cell ruptures, infecting other macrophages (Burza et al. 2018). After being ingested by a sandfly during a blood meal, amastigotes revert to flagellated promastigotes (Bates 2007).

Current treatment for leishmaniasis relies primarily on chemotherapy, using a limited number of drugs that present significant drawbacks (Abdellahi et al. 2022). One of the major concerns is the high toxicity of amphotericin B formulations, which are administered intravenously and often necessitate hospitalization (Sangshetti et al. 2015). Common adverse effects—such as fever, nausea, vomiting, nephrotoxicity, and cardiotoxicity—not only contribute to high discontinuation rates and poor patient adherence but may also accelerate the development of drug resistance (Sangshetti et al. 2015). Furthermore, treatment regimens typically last between 10 and 30 days and may require re‐treatment, compounding the financial burden (Berhe et al. 2024). For instance, the cost of amphotericin B deoxycholate varies widely across countries, ranging from less than USD 1 to as much as USD 171 per day (Berhe et al. 2024). Although liposomal amphotericin B offers a less toxic alternative, its substantially higher cost restricts its use in many low‐resource settings (Berhe et al. 2024).

Despite the introduction of miltefosine early in this century, the drug arsenal for leishmaniasis remains limited, primarily consisting of pentavalent antimonials, with amphotericin B and pentamidine as second‐line options (Kedzierski et al. 2009). Additionally, there is currently no available vaccine (Coutinho De Oliveira et al. 2020).

Recent research underscores the need for new treatment targets and innovative approaches, highlighting peptides as promising candidates through ongoing screening of natural and synthetic compounds (Robles‐Loaiza et al. 2021). Notably, in addition to the previously mentioned advantages, AMPs have a low likelihood of generating resistant strains due to their membrane‐disrupting mechanism, making peptide‐based therapies a viable option for leishmaniasis, though their effectiveness against *Leishmania* spp. requires further exploration (J. Li et al. 2017).

Limited studies have focused on the leishmanicidal effects of peptides, their structural characteristics, and current limitations. While Robles‐Loaiza et al. (2021) review examined around 50 articles on this topic, numerous experimental studies on AMPs against leishmanial infections have been published since, indicating the need for an updated scoping review on their sources, types, and efficacy. Therefore, our objective was to map the existing literature on the use of AMPs in the context of leishmaniasis, identifying the main findings and knowledge gaps on the topic addressed. To the best of our knowledge, this is the first review to focus on quantitative parameters to provide a data‐driven assessment of anti‐leishmanial AMPs, thereby supporting rational peptide design and complementing prior predominantly narrative‐focused reviews.

## 2 Materials & Methods

### 2.1 Protocol and Registration

**FIGURE 1:**
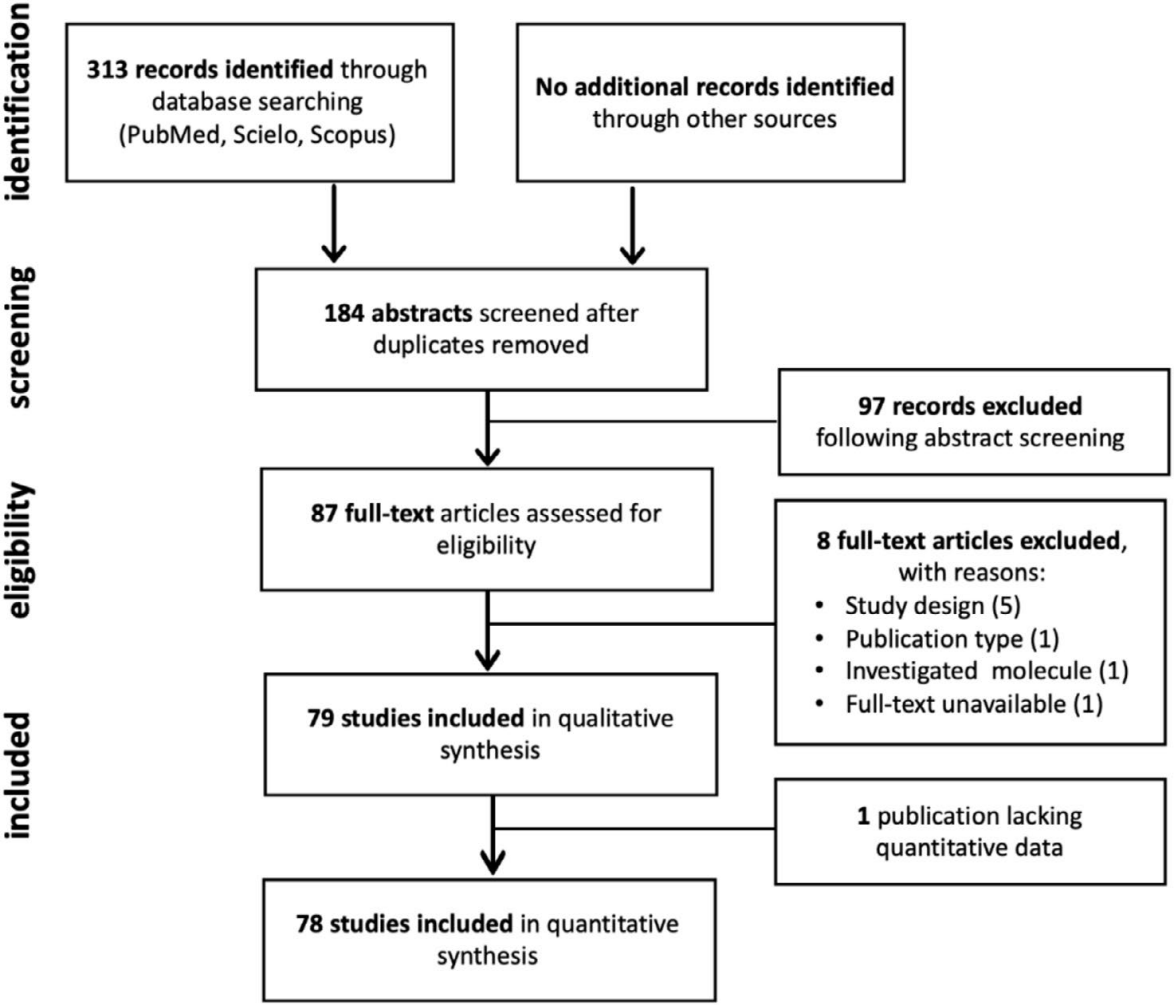
Flow diagram of literature search. PRISMA flow chart of literature search and selection process.

A scoping review was conducted in accordance with the Preferred Reporting Items for Systematic Reviews and Meta‐analysis Extension for Scoping Reviews (PRISMA‐ScR) (Figure 1) (Tricco et al. 2018). The final protocol was registered on the Open Science Framework platform on February 2, 2024 (https://doi.org/10.17605/OSF.IO/BMXC7).

### 2.2 Eligibility Criteria

The inclusion criteria encompassed antimicrobial peptides exhibiting potential leishmanicidal activity, explored through various methods such as in vivo and in vitro studies. No time limits were imposed, and we considered both published articles and pre‐prints to capture a wide range of findings. Reviews were excluded to focus on primary research. Furthermore, studies that focused exclusively on the effects of antimicrobial peptides on immune responses in experimental models, the natural human immune response, the natural immune response of vectors, in silico analyses, or solely on biological membranes were excluded. The publications were restricted to English‐language, excluding studies in other languages and those without full‐text availability.

### 2.3 Information Sources

A literature search was conducted using MEDLINE (PubMed), SciELO (Scientific Electronic Library Online), and Scopus databases in January 2024 to identify the relevant studies.

### 2.4 Search

The research was created by assembling terms reporting on leishmanial activity and antimicrobial peptides. The complete search strategies are presented in Table 1.

**TABLE 1:** Search strategy in four databases.

Data base	Search strategy
PubMed	leishman* [title/abstract] AND “antimicrobial peptide*”[title/abstract]
Scielo	(ab:(leishman$)) AND (ab:(antimicrobial)) AND (ab:(peptide$))
Scopus	TITLE‐ABS‐KEY (leishman*) AND TITLE‐ABS‐KEY (“ANTIMICROBIAL PEPTIDE*”)

### 2.5 Selection of Sources of Evidence

Search results collected in electronic databases were exported to the Rayyan platform (Rayyan.ai), where duplicate studies were removed. Two independent authors (M.E.V.O., O.H.P.G.) screened all titles and abstracts retrieved to identify eligible studies, and the differences were resolved by a third researcher (F.T.‐P.). If the title and abstract indicated that a study might be eligible, a full text was obtained. If the full text confirmed that the study met the eligibility criteria for this review, the study's data was extracted as follows.

### 2.6 Data Charting Process

Three independent reviewers (E.D.P.R., M.E.V.O., O.H.P.G.) performed the data extraction process using a standardized electronic form, which had been defined and agreed upon in advance. The extracted data were article identification, qualitative data fragments (characteristics of peptide‐based agents, dose applied, and toxicity assays), quantitative data (effective dose of each antileishmanial substance, toxicity), including selected cases when concentrations of peptide solutions were converted to μM by the reviewer. Peptide molecular weights were calculated from reported sequences using PeptideMass (ExPASy Bioinformatics Resource Portal, Swiss Institute of Bioinformatics) (Wilkins et al. 1999) and values originally reported in μg/mL were converted to μM using the formula: μM = (μg/mL × 1000)/MW (g/mol). Descriptions of the methods used, including study design, experimental model, sample sizes, and sample characteristics, were also extracted. The full list of data items is specified on Table S1.

Furthermore, the origin of the peptides was evaluated, with a classification based on their origin. Peptides were classified as “natural” when obtained from living sources and “synthetic” when synthesized through biotechnological methods, either with or without modifications to the amino acid sequences of the originally extracted natural peptides. Additionally, we assessed the living organisms from which these peptides were originally derived.

Recurring peptide sources and study methods were identified and categorized. Likewise, overarching themes across multiple studies were acknowledged and delineated. Data coding, aggregation, and charting were executed in Microsoft Excel, employing both extracted data and framework coding. The data underwent analysis using descriptive statistics. Following PRISMA‐ScR guidelines (Tricco et al. 2018), we opted not to conduct a critical appraisal analysis of the included studies.

## 3 Results

Initially, 313 records were identified through a database search, with no additional records found from alternative sources. After duplicate removal, 184 records remained. Following abstract screening, 97 records were excluded for not meeting the inclusion criteria, leaving 87 for full‐text evaluation. Of these, 8 were excluded due to inappropriate study design, publication type, non‐AMP molecules, or unavailability of the full text. The remaining 79 studies were included in the qualitative review, with 78 progressing to the quantitative analysis, as one focused solely on a nanosheet model. The study selection process and results are detailed in the PRISMA flow diagram (Figure 1).

### 3.1 Characteristics of Included Studies

AMPs were classified into two main categories based on their origin: “natural peptides”, directly isolated from living organisms, and “synthetic peptides”, produced through artificial synthesis. The synthetic group was further subdivided into two types: “purely synthetic peptides”, designed through computational approaches, and bioengineered peptides, which either replicate natural sequences or incorporate non‐natural modifications or substitutions. The bioengineered peptides were additionally categorized according to their source of inspiration into four subgroups: “reptile/amphibian”, “plant”, “microorganism”, and “arthropod”.

**FIGURE 2:**
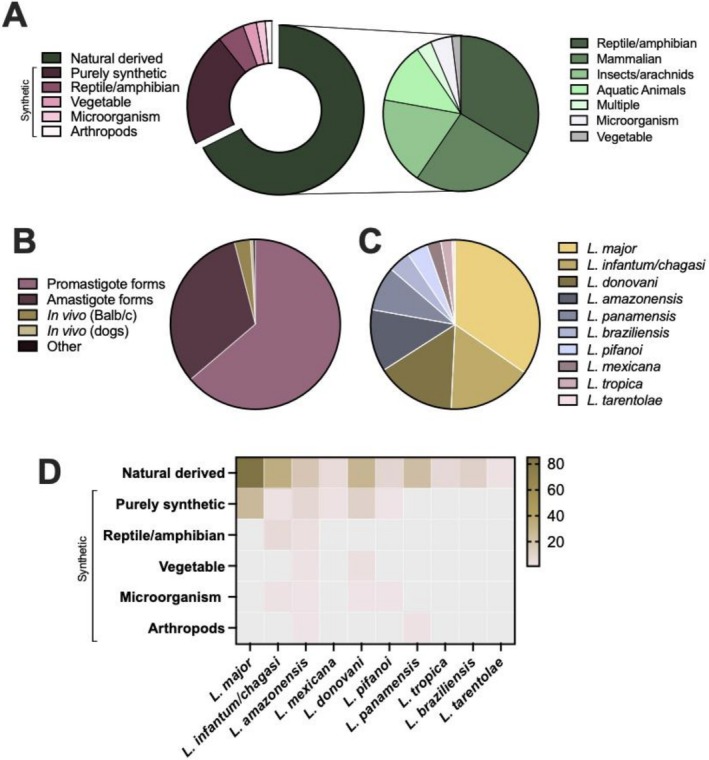
Overview of studies evaluating the effects of peptides in leishmaniasis treatment. (A) Distribution of studied peptides by source. AMPs were classified as natural (isolated from living organisms) or synthetic (artificially produced). Synthetic peptides were further divided into purely synthetic (computationally designed) and bioengineered (modified or mimicking natural sequences), the latter categorized by origin: reptile/amphibian, plant, microorganism, or arthropod. (B) Distribution of studies by experimental models tested. (C) Frequency of investigated *Leishmania* species. (D) Heatmap illustrating the distribution of studies across *Leishmania* species according to peptide origin.

Regarding the peptide source, the tested molecules included 157 bioengineered peptides, 49 purely synthetic peptides, 12 extracted from reptile or amphibian sources, 6 from plants, 3 from arthropods, and 4 from microorganisms (Figure 2A).

Concerning the experimental model used by the included works, out of the 79 included studies, 9 studies involved in vivo experiments, and one utilized a nanosheet model. The remaining studies were restricted to in vitro models, testing either amastigotes (*n* = 137) or promastigotes (*n* = 271) (Figure 2B).

Various Leishmania species were tested, primarily *L. major* (*n* = 114), followed by *L. infantum* (syn. *chagasi*) (*n* = 53), *L. donovani* (*n* = 50), *L. amazonensis* (*n* = 39), *L. panamensis* (*n* = 28), *L. braziliensis* (*n* = 14), *L. pifanoi* (*n* = 13), *L. mexicana* (*n* = 9), *L. tropica* (*n* = 7), and *L. tarentolae* (*n* = 2) (Figure 2C). Notably, some studies evaluated multiple peptides or tested more than one experimental model or *Leishmania* species. Overall, the most prevalent study design observed in the reviewed articles involved a naturally derived peptide tested on a strain of *L. major* , which appeared in more than 80 articles (Figure 2D).

**FIGURE 3:**
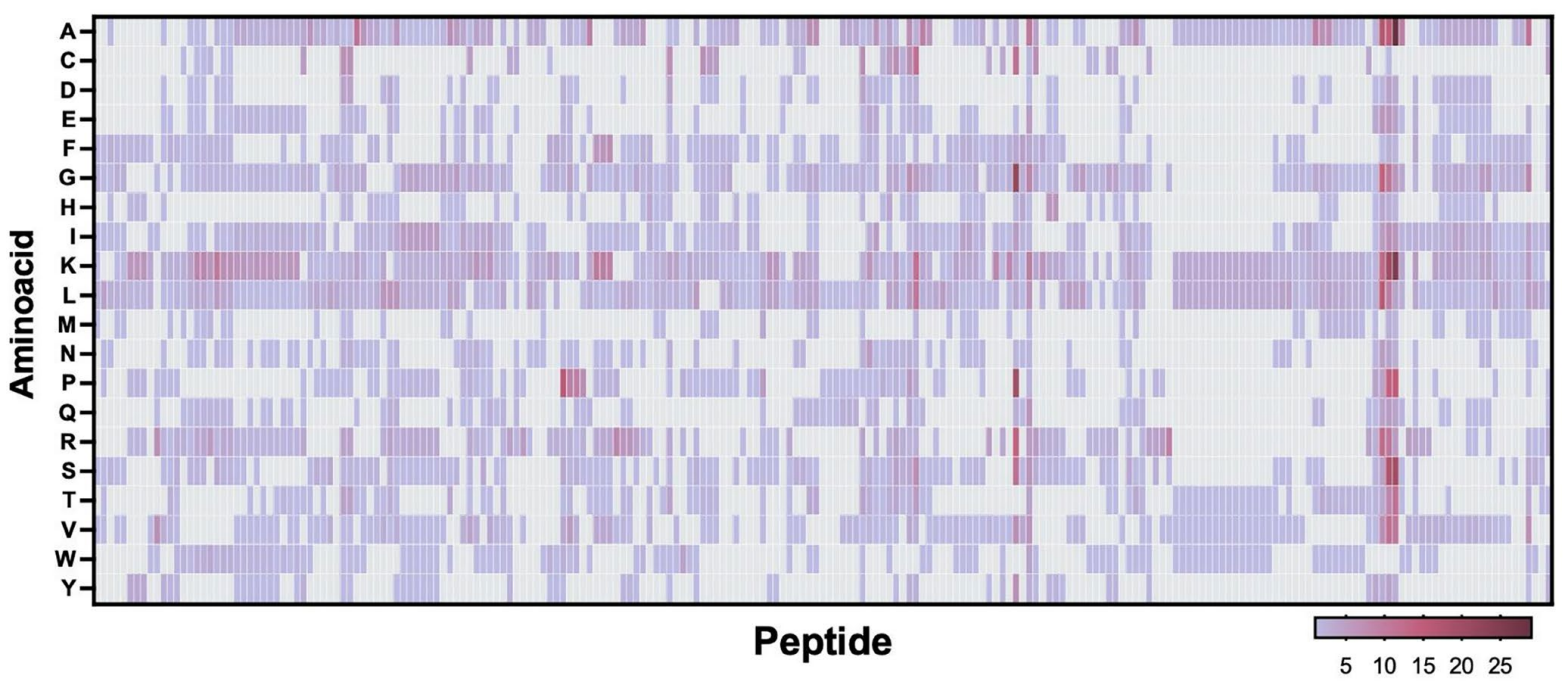
Heatmap of amino acids studied for their anti‐Leishmania properties. Alanine (A), cysteine (C), aspartic acid (D), glutamic acid (E), phenylalanine (F), glycine (G), histidine (H), isoleucine (I), lysine (K), leucine (L), methionine (M), asparagine (N), pyrrolysine (O), proline (P), glutamine (Q), arginine (R), serine (S), threonine (T), selenocysteine (U), valine (V), tryptophan (W), tyrosine (Y). The most frequent amino acids comprising the peptides were K, L, A, G, R, and I.

Finally, a heat map was built based on the amino acid composition of each peptide to reveal structural trends. The most prominent amino acids comprising the peptides were lysine (*n* = 825), leucine (*n* = 604), alanine (*n* = 576), glycine (*n* = 510), arginine (*n* = 417), and isoleucine (*n* = 368) (Figure 3).

The most promising peptides, selected primarily based on half maximal inhibitory concentration (IC50) and selectivity index (SI) values, are described in detail in the main text. Data on inactive or less active peptides are provided in Tables S2–S9.

### 3.2 Bioengineered Peptides

Peptides with non‐natural chemical modifications or derived from protein sequences have been identified across a broad range of organisms, each contributing distinct properties that may enhance their leishmanicidal potential. A total of 52 peptides were sourced from amphibians and reptiles, 43 from mammals, 29 from insects and arachnids, 7 from microorganisms, 19 from marine animals, 3 from plants, 1 from nematodes, and 3 from multiple sources (Figure 2A, Table S1).

Mammalian‐derived peptides were primarily inspired by molecules from *Bos taurus* (22 peptides), *Homo sapiens* (17 peptides), and *Camelus dromedarius* (3 peptides). Among these, peptides derived from bovine lactoferrin exhibited remarkable leishmanicidal activity, with LFampin–LFcin standing out as the most potent. This cryptopeptide combination demonstrated an IC50 of 1.7 μM against *L. donovani* promastigotes and 1.6 μM against axenic *L. pifanoi* amastigotes. The formation of a stable secondary structure is essential for a peptide's effective partitioning into its target, enhancing its interaction and overall efficacy (Adão et al. 2011). In LFampin–LFcin, the simple α‐peptide bond linking the two cryptopeptides promotes an optimized secondary structure, facilitating stronger interactions with *Leishmania* targets.

Insects and arachnids have been significant sources of antimicrobial peptides, with notable contributions from *Eumenes* spp. (6 peptides), *Apis* spp. (4 peptides), and *Hyalophora cecropia* (3 peptides). Additional species such as termites, triatomines, ants, and carpenter bees further enrich the diversity of insect‐derived AMPs. Among these, melittin from the venom of *Polistes* wasps demonstrated the highest potency, with an IC50 below 2.2 μM. However, its therapeutic development is hindered by its significant hemolytic and cytotoxic properties.

Among amphibian and reptile‐derived peptides, only one, BatxC, originated from reptiles, while the remaining 51 peptides came specifically from anurans. Among these peptides, Temp‐SHd showed action in two studies: in the study by Abbassi et al. (2013) against promastigotes of diverse *Leishmania* species and also against amastigotes of *L. infantum* with an IC50 6.7 μM and a SI no lower than 6 in this assay. Against the promastigotes, SI was sometimes close to 1, depending on the tested cells in the citotoxicity assays. In the study by André et al. (2020) Temp‐SHd showed activity against promastigotes of *L. infantum* (IC50: 16.5 μM and SI = 2.55), *L. brasiliensis* (17.9 μM and SI = 2.35), and *L. major* (14.6 μM and SI = 2.88). In vitro assays also showed good results for Cry3Aa‐DS1, with an IC50 of 0.67 μM and a SI = 81.61 for amastigotes of *L. amazonensis* and an IC50 of 0.30 μM and a SI = 182.27 for amastigotes of *L. donovani* (Yang et al. 2019).

Marine animals, particularly *Mytilus galloprovincialis* (6 peptides), *Litopenaeus vannamei* (4 peptides), and *Tachypleus tridentatus* (3 peptides), have been sources of promising peptides. Among marine‐derived peptides, Tachyplesin I emerged as the most effective against *Leishmania*, evaluated across three studies. It was the only marine‐derived peptide tested for IC50, exhibiting an IC50 of 18.6 μM and an LD50 of 9.3 μM, primarily against the amastigote form of *L. major* with exposure times ranging from 24 to 72 h. However, this peptide was inactive against *L. panamensis* . Despite its potential, Tachyplesin I demonstrated hemolytic or cytotoxic effects and lacked selectivity (SI = 1.2), limiting its viability as a therapeutic candidate (Löfgren et al. 2008; Lozano et al. 2014; Pérez‐Cordero et al. 2011).

A single study has investigated peptides derived from multiple sources, focusing on three proposed secondary sequences (Pr‐1, Pr‐2, and Pr‐3) of Protamine, a polycationic peptide with antimicrobial activity derived from various animal sperm (Pérez‐Cordero et al. 2011). These sequences were tested against *L. major* and *L. panamensis* , showing leishmanicidal activity exclusively against the latter (Pérez‐Cordero et al. 2011). Protamine also exhibited cytotoxic activity against human cells (Pérez‐Cordero et al. 2011).

HePC/AAM, a microorganism‐derived peptide, demonstrated strong activity with an IC50 of 0.3 μM against *L. infantum* amastigotes, suggesting its potential for therapeutic use (Fragiadaki et al. 2018). Plant‐derived peptides have not been tested for IC50, but they have shown leishmanicidal activity through growth inhibition of *L. amazonensis* promastigotes, with inhibition rates ranging from 33% to 96% over exposure times of 8–48 h (Souza et al. 2013, 2019). Only one nematode‐derived peptide, *Cecropin P1* from *Ascaris suum*, was analyzed. It was found to be inactive against both *L. panamensis* and *L. major* (IC50 > 14.97 μM) and exhibited high cytotoxicity with a selectivity index (SI) of 1 (Lozano et al. 2014).

The species most frequently tested across all categories was *L. major* , with 24 peptides derived from mammals and 25 from insects/arachnids, predominantly using the promastigote form of the parasite in studies that varied from 4 to 120 h of exposure. Amphibians and reptiles‐derived peptides were mainly tested in *L. infantum* (28 times). Marine‐derived peptides also primarily targeted *L. major* , while plant‐derived peptides focused on *L. amazonensis*. These variations in experimental conditions (such as the choice of parasite form (promastigote or amastigote) and exposure time) highlight the need for standardized testing protocols to compare the efficacy of these diverse peptides effectively.

Due to the challenge of designing stable structures that are also bioavailable at infection sites, only nine leishmanicidal peptides have reached in vivo trials, and none have advanced to clinical trials yet.

Campos‐Salinas et al. (2014) investigated the therapeutic potential of vasoactive intestinal peptide (VIP) derivatives against *L. major* infection in mice. Mice were subcutaneously injected with 10^4^ *L. major* metacyclic promastigotes in the left hind footpad, followed by administration of vehicle or peptides (1.5 nmol/mouse) three times a week for seven weeks, starting two weeks post‐infection (Campos‐Salinas et al. 2014). Disease progression was monitored by measuring inflammatory edema and lesion area, and parasite burden was assessed through a limiting dilution assay and histological analysis of the footpad, spleen, and lymph nodes. Both VIP derivatives significantly reduced the viability of *L. major* promastigotes, leading to decreased paw swelling, lesion size, and controlled clinical manifestations of the disease, including prevention of systemic parasite dissemination to immune and visceral organs, while VIP had no effect (Campos‐Salinas et al. 2014).

In a study by the same research group, UCNII demonstrated a protective effect in leishmaniasis in infected Balb/c mice, significantly reducing viable parasites in cutaneous lesions and decreasing lesions and necrosis caused by *L. major* . Administered subcutaneously in the infected footpad for seven weeks, starting two weeks post‐infection, UCNII also completely prevented the systemic spread of the pathogen to immune and visceral organs (Campos‐Salinas et al. 2013).

Abdossamadi, Seyed, et al. (2017) demonstrated the efficacy of human neutrophil peptide 1 (HNP‐1) against *L. major* in BALB/c mice. The mice were infected with promastigotes and divided into five groups, each receiving different peptide doses via footpad injections over three consecutive weeks. The results indicated that HNP‐1 both modulated the immune response and directly targeted the parasite, leading to significant reductions in parasitemia, histological inflammation in specific tissues, and increases in immune markers that contributed to controlling disease progression. In another experiment, Abdossamadi, Taheri, et al. (2017) used non‐pathogenic *L. tarentolae*, genetically engineered to secrete HNP‐1, to treat BALB/c mice infected with *L. major* . After three weeks of weekly injections (2 × 10^5^ cells/50 μL), real‐time PCR analysis revealed a significant decrease in *L. major* load in the lymph nodes of treated mice compared to the controls.

Yang et al. investigated the dermaseptin S1 peptide (DS1) and its conjugated Cry3Aa‐DS1 against *L. amazonensis* in BALB/c mice. Mice received the peptide via footpad injections every 4 days for a total of 6 times. The results indicated significantly greater reduction in lesion thickness and parasite burden for Cry3Aa‐DS1 compared to controls and to DS1 alone (in vitro results regarding this peptide were described in another paragraph above) (Yang et al. 2019).

### 3.3 Purely Synthetic Peptides

In regard to synthetic peptides, 49 studies were identified, testing 45 different peptides against promastigote and amastigote forms of different *Leishmania* species. Peptide CM11 appears in 5 different studies; Esmaeilifallah et al. (2023) found that the peptide starts its leishmanicidal activity at the 24 h mark, with an IC50 of 7.56 μM (SI = 0.99) for the amastigote form of *L. major* and 7.03 μM (SI = 1.06) for the promastigote form of the same species (Esmaeilifallah et al. 2023).

That same study also conducted in vivo experiments on Balb/c mice, which found parasite burden in the spleen, lymph nodes, and liver to be significantly inferior compared to negative control, and in regard to toxicity, all animals survived the 90 days of experiment (Esmaeilifallah et al. 2023).

Aqeele et al. (2019, 2021), however, found CM11 to be inactive against both forms of *L. major* , testing 8 μM/mL of the peptide alone and in combination with 10 and 20 μM/mL of curcumin (no synergic effect was observed).

Overall, CM11's effect seems to be significant, for it was also found to have a leishmanicidal effect in a study by Khalili et al. (2019) with IC50s of 9.02 and 6.92 μM, respectively for amastigote and promastigote forms of *L. major*. The same author had conducted a similar study in 2018, with intra macrophage amastigotes of *L. major*, which showed an IC50 of 9.58 μM (Khalili et al. 2018).

The remaining peptides were only tested by one study each, and those with the most promising results are further described in this section. Those peptides found to be inactive or active but with higher IC50s are mentioned in Table S2.

In Costa et al. (2023), TSHa, and its conjugated form GVL1‐TSHa, showed significant activity against *L. amazonensis* and *L. mexicana* . The first variant's IC50s varied from 6.3 to 8.0 μM between amastigotes and promastigotes of both leishmania species. When the peptide was conjugated with GVL1, however, an IC50 as low as 0.33 μM was observed, with a selectivity index of 6060 (*L. amazonensis* amastigotes) (Costa et al. 2023).

Costa also tested p‐Bt and its conjugated forms (GVL1)‐p‐Bt and (GVL1)2‐p‐Bt against amastigote and promastigote forms of *L. amazonensis*. (GVL1)‐p‐Bt had the most impressive results, with IC50s of 0.9 μM and 1.1 μM for amastigotes and promastigotes respectively, and a SI of 2222 (calculated for amastigote forms) (Costa et al. 2023).

Corman et al. (2022) tested several bacteriocins on *L. donovani* amastigotes (Syn‐enterocins, Syn‐safencins, and Syn‐larvacin). Most of the peptides had better results on axenic amastigotes as opposed to intracellular amastigotes, with IC50 values ranging from 0.27 μM (SI = 74.07) to 0.94 μM (SI = 21.19) for the first, and from 2.04 μM (SI = 9.82) to 23.67 μM (SI = 0.85) for the latter (Corman et al. 2022).

Radzishevsky et al. (2005) tested peptide P (ALWKTLLKKVLKA) and 14 of its conjugated forms, all of which were found to induce cellular lysis on *L. major* promastigotes, with MLCs varying from 3.12 μM for C14‐P, to 25 μM for NC2‐P (Radzishevsky et al. 2005).

### 3.4 Reptile/Amphibian

Among the 12 identified reptile or amphibian peptides, only 5 were considered to have leishmanicidal activity. Abbassi et al. (2008) tested 3 different variations of Temporin peptide (from *Pelophylax saharica*) against *L. infantum* promastigotes and axenic amastigotes. Temporin‐1Sa was the only one to exhibit leishmanicidal activity, with an IC 50 of 18.1 μM against promastigotes (SI = 1.38) and 22.8 μM against amastigotes (SI = 1.09) (Abbassi et al. 2008).

Peptides from *Phyllomedusa* spp. were also tested by Pinto et al. (2013) and Brand et al. (2013). The first author studied four *P. nordestina* peptides, one of which (Phylloseptin 7) was found active against *L. infantum* promastigotes (IC50 = 10.06 μM and SI = 3.421) (Pinto et al. 2013). In the study by Brand et al. (2013) DS 01 and DShypho 01 (from *P. hypochondrialis* ) were also found to have leishmanicidal activity. There was no calculated IC50, but the authors found protozoan cell population to be reduced to a non‐detectable level at peptide concentrations close to 64 μM/L after 2 and 6 h of incubation.

More recently, Allane et al. (2018) tested Disintegrin from *Cerastes cerastes* against *L. infantum* promastigotes and found an estimated mortality of 87% at 0.01 mg/mL. The remaining peptides were found to be inactive against Leishmania species, as shown in Table 2.

**TABLE 2:** Reptile‐ or amphibian‐derived peptides studied against *Leishmania* spp.

Peptide	Species of origin	Species	Form	Findings	IC50 (μM)	SI	Author, year
Temporin‐1Sa	*Pelophylax saharica*	*L. i*	Promastigote	Leishmanicidal activity	18.1	1.38	Abbassi et al. (2008)
			Amastigote		22.8	1.09	
Temporin 1‐Sb	*Pelophylax saharica*	*L. i*	Promastigote/amastigote	Inactive	NI	NI	
Temporin 1‐Sc	*Pelophylax saharica*	*L. i*	Promastigote/amastigote	Inactive	NI	NI	
Dermaseptin 4	*Phyllomedusa nordestina*	*L. i*	Promastigote	Inactive	NI	NI	Pinto et al. (2013)
Dermaseptin 1	*Phyllomedusa nordestina*	*L. i*	Promastigote	Inactive	NI	NI	
Phylloseptin 7	*Phyllomedusa nordestina*	*L. i*	Promastigote	Leishmanicidal activity	10.1	3.42	
Phylloseptin	*Phyllomedusa nordestina*	*L. i*	Promastigote	Inactive	NI	NI	
Bax3k	*Bothrops atrox*	*L. a*	Promastigote	Inactive	NI	NI	da Silva Caldeira et al. (2021)
Bj3k	*Bothrops jararacussu*	*L. a*	Promastigote	Inactive	NI	NI	
Disintegrin_Cc	*Cerastes cerastes*	*L. i*	Promastigote	Leishmanicidal activity	NI	NI	Allane et al. (2018)
DS 01	*Phyllomedusa hypocondrialis*	*L. a*	Promastigote	Leishmanicidal activity	NI	NI	Brand et al. (2013)
DShypho 01	*Phyllomedusa hypocondrialis*	*L. a*	Promastigote	Leishmanicidal activity	NI	NI	

Abbreviations: *L.a*, *L. amazonensis*; *L.i*, *L. infantum*; NI, not informed.

### 3.5 Plants

Plants peptides weren't as prevalent in the studies, with 6 different forms tested on different species of leishmania. Souza et al. (2013) tested Vu‐Def, from *Vigna unguiculata*, which inhibited the growth of 50% of *L. amazonensis* promastigotes at the 24 h mark, and 54% at the 48 h mark (Souza et al. 2013).

PvD1, a peptide from *Phaseolus vulgaris* , also showed promising results. At concentrations of 55.06 μM and 110.13 μM it was able to inhibit proliferation of *L. amazonensis* promastigotes by 70% and 87% in 24 h and 89% and 96.5% at 48 h (Do Nascimento et al. 2015).

Lastly, Berrocal‐Lobo et al. (2009), tested 4 different peptides against *L. donovani* promastigotes and amastigotes. Thionin alfa 1 was active against promastigotes, with an IC50 of 0.2 μM; it also exhibited some activity against amastigotes, but this form was more resistant, with a LC50 of 46.3 μM. Pseudothionin‐St1 also influenced leishmania promastigotes but had a much higher IC50 of 33.4 μM. The other peptides tested in this study (LTP2 alpha 1 isoform and snakin 1) were not active (Berrocal‐Lobo et al. 2009).

### 3.6 Microorganism as Peptide Source

The reviewed studies emphasize the potential of peptides derived from microorganisms in targeting both amastigote and promastigote forms of *Leishmania* species (Table 3). Fodor et al. (2023) investigated the effects of peptides produced by bacteria of *Xenorhabdus* species, specifically from the cell‐free culture media of *X. budapestensis* , *X. szentirmaii* , and *X. innexii*, against *L. amazonensis*. They found that the culture media contained peptides capable of killing the parasite without exhibiting toxicity towards J774 macrophages. However, the specific peptides responsible for this activity were not identified, and their inhibitory concentrations were not determined (Fodor et al. 2023).

**TABLE 3:** Peptides from microorganisms tested against *Leishmania* spp.

Peptide	Species of origin	Species	Form	IC50 (μM)	SI	Author, year
Mixture in cell‐free culture media	*Xenorhabdus* spp.	*L. a*	Promastigote	NI	NI	Fodor et al. (2023)
Antiamoebin (AAM)	*Emericellopsis synnematicola*	*L. i*	Amastigote	7.5	5.37	Fragiadaki et al. (2018)
			Promastigote	8.7	4.63	
Suzukacillin (SZ)	*Trichoderma viride*	*L. i*	Amastigote	7.6	2.96	
			Promastigote	8.2	2.74	
AS‐48	*Enterococcus faecalis*	*L. d*	Promastigote	3.9	NI	Abengózar et al. (2017)
		*L. p*	Amastigote	9.4	NI	

Abbreviations: *L.a*, *L. amazonensis*; *L.d*, *L. donovani* ; *L.i*, *L. infantum*; *L.p*, *L. pifanoi*; NI, not informed.

In the same way, Abengózar et al. (2017) studied the peptide AS‐48 from bacteria *Enterococcus faecalis* against *L. donovani* and *L. pifanoi*. The results showed action for both species, with IC50 of 3.9 μM for *L. donovani* in the promastigote form and 9.4 μM for *L. pifanoi* axenic amastigote. No data on AS‐48 in host cells were provided (Abengózar et al. 2017).

Fragiadaki et al. (2018) assessed peptides from the fungus *Emericellopsis synnematicola* against *L. infantum*. The authors reported an IC50 of 7.5 μM and an SI of 5.37 for amastigotes, while for promastigotes, the IC50 was 8.7 μM with an SI of 4.63. Additionally, the authors tested peptides from another fungus, *Trichoderma viride*, against *L. infantum*, revealing an IC50 of 7.6 μM (SI = 2.96) for amastigotes and 8.2 μM (SI = 2.74) for promastigotes (Fragiadaki et al. 2018).

### 3.7 Arthropods

Among insect and arachnid peptides, only 2 studies were found. Patiño‐Márquez et al. (2018a, 2018b) tested 2 peptides from *Galleria mellonella* moth for their activity against *L. panamensis* promastigotes. Anionic peptide 2 showed a decrease in parasite viability of 45% at a concentration of 10 μM. Whereas Cecropin‐D had a 43% decrease at a concentration of 100 μM (Patiño‐Márquez et al. 2018a, 2018b).

Gomesin, from the spider *Acanthoscurria gomesiana* , was studied by Silva Jr. et al. (2000). Though the focus of the study wasn't leishmania, it was tested against *L. amazonensis* promastigotes, and the authors found a 50% reduction in parasite viability at a 2.5 μM concentration of Gomesin.

In summary, available data on the leishmanicidal activity of arthropod peptides remain scarce; therefore, further studies will provide more robust evidence regarding their leishmanicidal potential and mechanisms of action.

## 4 Discussion

Leishmaniasis remains a neglected tropical disease with profound global health and socioeconomic impact. Despite extensive research, no effective vaccine exists, and current treatments are limited by high toxicity, cost, and rising drug resistance. Diagnostic delays and increased migration further expand endemic areas and complicate control efforts. These challenges have prompted the pursuit of novel, safer, and more accessible therapies. Among these, AMPs have gained considerable attention in recent years due to their demonstrated efficacy, standing out alongside other strategies such as nanocarrier‐based delivery, drug repurposing, and natural product exploration.

By systematically analyzing selectivity indices across studies, this review provides a practical data‐driven framework to guide the design and optimization of anti‐leishmanial AMPs, complementing prior narrative‐focused reviews. While Robles‐Loaiza et al. (2021) compiled a valuable database of over 140 unique peptide sequences, our analysis identified 231 distinct peptides, reflecting both the expansion of available data and the continued interest in AMPs as promising therapeutic candidates for leishmaniasis (Robles‐Loaiza et al. 2021). In contrast to previous works, we systematically report detailed metrics such as IC_50_ values and selectivity indices (SI), offering a more robust assessment of peptide efficacy and safety in order to guide future studies. Additionally, by including studies published through 2024, our review captures the most recent advances in the field.

AMPs primarily kill *Leishmania* by disrupting the parasite's membranes (Arcisio‐Miranda et al. 2008; Coorens et al. 2017; Järvå et al. 2018; Kvansakul et al. 2016; Scheenstra et al. 2019; Sepehri et al. 2020). By rapidly permeabilizing and depolarizing the parasite's plasma membrane, they cause leakage of intracellular contents and ultimately induce cell death, a mechanism exemplified by temporin‐SHa and its analogs (Mangoni et al. 2005; Raja et al. 2017). High‐resolution imaging and biophysical studies reveal that these peptides insert themselves into the lipid bilayer, disturbing its structure in a detergent‐like manner (Bahar and Ren 2013). This strategy proves highly effective against both promastigote and intracellular forms of the parasite (El‐Dirany et al. 2021).

Beyond direct membrane lysis, certain AMPs can also initiate apoptosis‐like processes at higher concentrations, including mitochondrial depolarization and DNA fragmentation (Mijiddorj et al. 2018; Pitale et al. 2020; Souza et al. 2018, 2019). Specifically, the non‐ribosomal peptaibols Antiamoebin and Suzukacillin induce mitochondrial dysfunction in *L. infantum*, characterized by membrane depolarization, excessive reactive oxygen species (ROS) production, calcium (Ca^2+^) imbalance, and mitochondrial swelling (Fragiadaki et al. 2018).

Immunomodulation is another mechanism by which AMPs exert anti‐leishmanial effects; for instance, cathelicidin enhances the immunomodulatory potential of Amphotericin B against *Leishmania* through a Toll‐like receptor 2 (TLR2)/vitamin D receptor (VDR)‐dependent mechanism (Das et al. 2017). Some peptides may even present additional intracellular effects, such as interfering with nucleic acids or protein synthesis, though these mechanisms remain less well defined in *Leishmania* than in bacteria (Zahedifard and Rafati 2018). Crucially, these peptides carry a low risk of resistance development, positioning them as promising candidates for novel anti‐leishmanial therapies in the face of growing drug resistance and the limitations of current treatments (Croft et al. 2006; Ponte‐Sucre et al. 2017).

Among the *Leishmania* species evaluated, *L. major* predominates, with roughly twice as many experiments in comparison to other species. This can be explained by a combination of methodological and geopolitical factors. *L. major* is a well‐established model for cutaneous leishmaniasis, supported by standardized in vitro systems and widely used animal models that provide reproducible and well‐characterized immune responses (Abdossamadi, Taheri, et al. 2017; Campos‐Salinas et al. 2013; El‐Dirany et al. 2022; Sacks and Noben‐Trauth 2002). Its relative ease of cultivation and extensive availability of experimental tools have further contributed to its widespread use in preclinical research (Khalili et al. 2019; Lynn et al. 2011). Additionally, research capacity and funding for neglected tropical diseases may contribute to the uneven distribution of studies across *Leishmania* species. Evidence suggests that research output often does not necessarily reflect the geographic burden of disease (Fonseca et al. 2020; Ramos et al. 2013).

Despite the practical advantages of using *L. major* , the assumption that it serves as a universal proxy for the genus is challenged by the fact that AMP susceptibility varies across species. The human neuropeptide urocortin II, present in epithelial and mucosal barriers, was active against *L. major* , *L. tropica* , and *L. infantum*, but inactive against *L. donovani* and *L. mexicana* (Campos‐Salinas et al. 2013), illustrating the limits of single‐species testing. Consistently, a comparative transcriptional study of five *Leishmania* species exposed to trivalent antimony found no shared differentially expressed gene, with resistance driven by species‐specific orthologs or “self‐genes” (Medina et al. 2021). Even within *L. donovani* populations, resistance markers vary by genetic background, producing distinct profiles under the same drug pressure (Decuypere et al. 2012). Together, these findings highlight substantial variability that may affect peptide efficacy, while cross‐species comparisons remain limited and no genome‐level advantage of *L. major* has been demonstrated (Medina et al. 2021).

In this context, given that species identification in clinical practice is often unavailable in endemic settings, where treatment is frequently initiated without molecular confirmation (de Vries and Schallig 2022), future research should prioritize the development and testing of broad‐spectrum, polyvalent peptides. It is recommended that studies include multiple species reflecting the epidemiological context to ensure translational relevance.

With regard to the nature of the peptides tested, in this review, we observed a marked discrepancy in efficacy between synthetic and naturally derived peptides. Bioengineered peptides exhibited higher SI values and lower IC50s compared to natural‐derived peptides, with an even greater difference when strictly natural peptides were considered. Notably, some synthetic peptides achieved SI values as high as 6000, indicating a remarkable ability to selectively target *Leishmania* spp. while sparing host cells. This difference may be attributed to multiple factors; our main hypothesis is that synthetic peptides can be precisely designed, optimizing amino acid sequences and biochemical structures to maximize leishmanicidal activity while minimizing cytotoxicity toward host cells (Lima et al. 2021).

Despite the promising features of natural and synthetic AMPs, there are several limitations that must be addressed. For example, the activity of many AMPs is significantly reduced or even completely abolished in the presence of physiological concentrations of salts or other biological compounds (Xu et al. 2023). Moreover, some AMPs are degraded by serum proteases, which greatly shortens their in vivo half‐life (Starr and Wimley 2017). Additionally, due to their broad and non‐specific mechanism of action, AMPs may exhibit some degree of cytotoxicity at therapeutic concentrations (Casciaro et al. 2020). However, these limitations may be less critical in the treatment of topical conditions such as cutaneous leishmaniasis, where these issues could be more easily managed (El‐Dirany et al. 2021).

To address the challenges associated with AMPs, various strategies have been explored. Some research teams have focused on enhancing the efficiency and activity of these peptides through chemical modifications, including glycosylation and lipidation (Bellavita et al. 2023). Others have reduced the size of drug carriers to the nanometric scale, enhancing their ability to be internalized by cells and to reach specific intracellular locations (Faya et al. 2018). Nanosystems composed of liposomes, dendrimers, solid‐core nanoparticles, or carbon‐based carriers have been developed to encapsulate AMPs, offering a promising platform to enhance their delivery to parasite cells (Faya et al. 2018).

Recent research has built on the framework outlined in the *2009 Drug Screening for Kinetoplastid Diseases* manual, which recommended that antileishmanial candidates target intramacrophagic *L. donovani* amastigotes (IC50 ≤ 10 μM) with at least 20‐fold selectivity over mammalian cells (Ioset et al. 2009). Since then, compounds such as guanidine‐functionalized AMPs (Costa et al. 2023) and minimal AS‐48 bacteriocin homologs (Corman et al. 2022) and TSHa‐type temporins (Costa et al. 2023) have achieved substantially lower IC50 values while retaining minimal cytotoxicity. These advances highlight a shift toward more rigorous development standards that prioritize not only in vitro potency but also host safety, pharmacokinetics, and in vivo efficacy. Consequently, the 2009 benchmarks now serve mainly as a reference, increasingly surpassed by newer and more potent candidates.

Another limitation to the clinical potential of AMPs in treating infections such as leishmaniasis is their poor metabolic stability and susceptibility to rapid degradation by serum proteases and other proteolytic enzymes (Al Musaimi et al. 2022; Lombardi et al. 2025; Wątły et al. 2021). These biomolecules typically exhibit short half‐lives, often less than 30 min, and low bioavailability because human and pathogenic endo‐ and exo‐peptidases transform high‐molecular‐weight peptides into inactive fragments (Lombardi et al. 2025; Wątły et al. 2021). To address these challenges, the development of peptidomimetics (modified natural peptides designed to retain biological activity while exhibiting improved chemical properties) has emerged as a widely utilized strategy in drug discovery (Al Musaimi et al. 2022; Lombardi et al. 2025; Wątły et al. 2021).

In this sense, various structural modifications can be employed to create peptidomimetic variants with enhanced proteolytic stability for potential use in leishmaniasis therapy. For example, the incorporation of D‐amino acids is highly effective because they rarely act as substrates for endogenous proteases, thereby significantly increasing a peptide's half‐life (Al Musaimi et al. 2022; Lombardi et al. 2025; Wątły et al. 2021). Other common strategies include the use of beta‐amino acids, which are metabolically more stable than standard alpha‐amino acids, and the integration of unusual amino acids (UAAs) that favorably influence metabolic stability and proteolytic resistance (Lombardi et al. 2025; Wątły et al. 2021). Furthermore, localized backbone modifications can obstruct protease binding by rigidifying the peptide structure and stabilizing its bioactive conformation (Al Musaimi et al. 2022; Lombardi et al. 2025; Wątły et al. 2021). These diverse peptidomimetic strategies have been successfully deployed across various medical fields, including cancer and infectious disease research, and provide a robust framework for optimizing AMPs for clinical applications (Lombardi et al. 2025).

In addition, a significant limitation in current research is the low number of in vivo studies. While in vitro experiments provide valuable insights, they do not replicate the complexities of host–parasite interactions, limiting our understanding of pharmacokinetics, toxicity, and the overall therapeutic potential of AMPs (Hanson et al. 2019). Additionally, most studies focus on *L. major* and typically examine only the amastigote or promastigote forms of the parasite. Expanding research to include a broader range of *Leishmania* species and life cycle stages would provide a more comprehensive evaluation of the therapeutic potential of AMPs.

In summary, this scoping review found that synthetic AMPs exhibit more potent antileishmanial activity than peptides derived directly from natural sources. While AMPs hold significant promise as therapeutic agents for leishmaniasis, challenges such as susceptibility to degradation, cytotoxicity at therapeutic doses, and the need for improved selectivity remain. In this sense, ongoing research into structural modifications, combination therapies with conventional drugs, and advanced delivery systems is paving the way for the clinical application of AMP‐based treatments. It is noteworthy that in vivo studies are still scarce, which is vital for validating the therapeutic efficacy and safety of potential drugs. The future of AMP therapy for leishmaniasis, particularly cutaneous leishmaniasis, looks promising, with the potential for clinical approval in the coming years.

## 5 Conclusions

Antimicrobial peptides represent a highly promising avenue for the development of new treatments against *Leishmania* sp., combining potent activity and low resistance potential with advances in peptide design and delivery systems. Nonetheless, challenges such as loss of activity in physiological environments, limited in vivo validation, and potential cytotoxicity must still be addressed to ensure their safe and effective translation into clinical practice.

## Author Contributions

**Ellen Dyminski Parente Ribeiro:** data curation, methodology, writing – review and editing, writing – original draft, investigation. **Maria Eduarda da Veiga Oliveira:** writing – review and editing, writing – original draft, data curation, methodology, investigation. **Otávio Henrique Pradi Guenther:** investigation, methodology, data curation, writing – review and editing, writing – original draft. **Fernanda Tomiotto‐Pellissier:** supervision, project administration, funding acquisition, writing – review and editing, conceptualization, resources, methodology.

## Funding

This study was partially financed by the Coordenação de Aperfeiçoamento de Pessoal de Nível Superior (CAPES). The authors acknowledge the support of SETI/Fundação Araucária, which provided a research productivity fellowship to F.T.‐P. (Public Call 23/2023; PD&I Agreement 269/2025) and an undergraduate research fellowship to E.D.P.R.

## Conflicts of Interest

The authors declare no conflicts of interest.

## Supporting information


**Table S1:** Data items collected during data extraction.
**Table S2:** IC50 and Selectivity Indices (SI > 1) of bioengineered peptides active Against Leishmania promastigotes.
**Table S3:** IC50 values and Selectivity Indices (SI) of bioengineered peptides exhibiting leishmanicidal activity against amastigotes.
**Table S4:** IC50 and Selectivity Indices (SI > 1) of purely synthetic peptides active Against Leishmania amastigotes.
**Table S5:** IC50 values and Selectivity Indices (SI) of purely synthetic peptides exhibiting leishmanicidal activity against axenic amastigotes.
**Table S6:** IC50 and Selectivity Indices (SI > 1) of purely synthetic peptides active Against Leishmania promastigotes.
**Table S7:** IC50 values and Selectivity Indices (SI) of reptile‐ or amphibian‐derived peptides exhibiting leishmanicidal activity.
**Table S9:** IC50 values and Selectivity Indices (SI) of microorganism‐derived peptides exhibiting leishmanicidal activity.

## Data Availability

The data that supports the findings of this study are available in the Supporting Information of this article.
