# Supplementary material for: Peptides as a Therapeutic Alternative Against Leishmaniasis: A Scoping Review
Source: Chem Biol Drug Des. 2026 May 18;107:e70314. doi: 10.1111/cbdd.70314 (PMC13184368; doi:10.1111/cbdd.70314)
Supplement: Supplementary file 1 — Table S1: Data items collected during data extraction. Table S2: IC50 and Selectivity Indices (SI > 1) of bioengineered peptides active Against Leishmania promastigotes. Table S3: IC50 values and Selectivity Indices (SI) of bioengineered peptides exhibiting leishmanicidal activity against amastigotes. Table S4: IC50 and Selectivity Indices (SI > 1) of purely synthetic peptides active Against Leishmania amastigotes. Table S5: IC50 values and Selectivity Indices (SI) of purely synthetic peptides exhibiting leishmanicidal activity against axenic amastigotes. Table S6: IC50 and Selectivity Indices (SI > 1) of purely synthetic peptides active Against Leishmania promastigotes. Table S7: IC50 values and Selectivity Indices (SI) of reptile‐ or amphibian‐derived peptides exhibiting leishmanicidal activity. Table S9: IC50 values and Selectivity Indices (SI) of microorganism‐derived peptides exhibiting leishmanicidal activity. [file CBDD-107-e70314-s001.docx]

**SUPPLEMENTARY MATERIAL**

**Table** S**1 - Data items collected during data extraction.**

| **Data item** | **Description** |
| --- | --- |
| peptide name | collected exactly as written in original paper |
| origin/class | authors classified peptides as: synthetic, natural sources or naturally derived (including synthesized peptides with an identical sequence to natural ones) |
| species of origin | if not specified in original paper a complementary search was made |
| aminoacid sequence | if not specified in original paper a complementary search was made |
| conjugated | collected exactly as written in original paper |
| leishmania species | collected exactly as written in original paper |
| parasite form | collected exactly as written in original paper, later simplified to either promastigote, amastigote or in vivo |
| results | collected exactly as written in original paper |
| IC50 | collected exactly as written in original paper |
| other statistical measurement | collected exactly as written in original paper |
| time response | collected exactly as written in original paper |
| cytotoxicity | collected exactly as written in original paper |
| selectivity index | if not specified in original paper, authors calculated using (cytotoxicity/IC50) |
| additional data | any relevant information from the study that wasn’t included in previous categories |
| Author, year, DOI and url | collected exactly as written in original paper |

**Peptides with available IC₅₀ and CC₅₀ data were compiled into tables, along with their selectivity index (SI), either as reported in the original studies or calculated by the authors. For consistency, values expressed in µg/mL were converted to µmol/L. The peptides were then organized by source—synthetic, bioengineered, plant‑derived, microorganism‑derived, reptile/amphibian‑derived, or arthropod‑derived—and listed in decreasing order of SI. Each entry also indicates the peptide form tested and the *Leishmania* species evaluated.**

**Table S2 – IC₅₀ and Selectivity Indices (SI > 1) of bioengineered peptides active Against *Leishmania* promastigotes.**

| **Peptide** | **Species of origin** | **Species** | **IC50 (μM)** | **SI *** | **Author, year** |
| --- | --- | --- | --- | --- | --- |
| **Mammals** |  |  |  |  |  |
| **TAP** | *Bos taurus* | *L. m* | 5.7 | 2.2 | Lozano et al, 2014 |
| **TAP** | *Bos taurus* | *L. p* | 11.3 | 1.1 | Lozano et al, 2014 |
| **Insects/Arachnids** |  |  |  |  |  |
| **Melittin** | *Polystes sp. HQL-2001* | *L. m* | < 2.2 | 8 | Lozano et al., 2014 |
| **Melittin** | *Polystes sp. HQL-2002* | *L. p* | 2.7 | 6.6 | Lozano et al., 2014 |
| **MP-8** | *Vespa magnifica* | *L. m* | 10.7 | 3.1 | Lozano et al., 2014 |
| **MP-8** | *Vespa magnifica* | *L. p* | 18.1 | 1.9 | Lozano et al., 2014 |
| **Polystes MA** | *Polystes jadwigae* | *L. m* | 25.2 | 1.2 | Lozano et al., 2014 |
| **Marine Animals** |  |  |  |  |  |
| **Tachyplesin I** | *Tachypleus tridentatus* | *L. m* | 18.6 | 1.2 | Lozano et 2014 |
| **Amphibians/Reptiles** |  |  |  |  |  |
| **Temporin-SHa** | *Rana temporaria* | *L. b* | 7 | 80 | Raja et al. 2017 |
| **Temporin [K3]SHa** | *Rana temporaria* | *L. b* | 5 | 71.6 | Raja et al. 2017 |
| **Temporin [K3]SHa** | *Rana temporaria* | *L. a* | 8 | 44.8 | Raja et al. 2017 |
| **Temporin-SHa** | *Rana temporaria* | *L. m* | 13 | 43.1 | Raja et al. 2017 |
| **Temporin-SHa** | *Rana temporaria* | *L. a* | 13 | 43.1 | Raja et al. 2017 |
| **Temp-SHd** | *Pelophylax saharicus* | *L. t* | 13.9 | 42.7 | Abbassi et al. 2013 |
| **Temp-SHd** | *Pelophylax saharicus* | *L. a* | 14.1 | 42.1 | Abbassi et al. 2013 |
| **Temp-SHd** | *Pelophylax saharicus* | *L. m* | 14.6 | 40.7 | Abbassi et al. 2013 |
| **Temporin-SHa** | *Rana temporaria* | *L. i* | 13.1 | 40 | Raja et al. 2017 |
| **Temporin [K3]SHa** | *Rana temporaria* | *L. i* | 9 | 39.8 | Raja et al. 2017 |
| **Temp-SHd** | *Pelophylax saharicus* | *L. i* | 16.5 | 36 | Abbassi et al. 2013 |
| **Temporin [K3]SHa** | *Rana temporaria* | *L. i* | 9.1 | 35.8 | Raja et al. 2017 |
| **Temporin [K3]SHa** | *Rana temporaria* | *L. m* | 10 | 35.8 | Raja et al. 2017 |
| **Temp-SHd** | *Pelophylax saharicus* | *L. b* | 17.9 | 34.7 | Abbassi et al. 2013 |
| **Temporin-SHa** | *Rana temporaria* | *L. i* | 17.1 | 31.1 | Raja et al. 2017 |
| **Bombinin** | *Bombina maxima* | *L. m* | <2.5 | 8 | Lozano et al. 2014 |
| **Temporin-SHe** | *Pelophylax saharicus* | *L. i* | 4.6 | 3.7 | André et al. 2020 |
| **Temporin-SHd** | *Pelophylax saharicus* | *L. m* | 14.6 | 2.9 | André et al. 2020 |
| **Temporin-SHd** | *Pelophylax saharicus* | *L. i* | 16.5 | 2.6 | André et al. 2020 |
| **Temporin-SHd** | *Pelophylax saharicus* | *L. b* | 17.9 | 2.4 | André et al. 2020 |
| **Dermaseptin S1** | *Phyllomedusa bicolor* | *L. p* | 7.1 | 2 | Lozano et al. 2014 |
| **Dermaseptin S1** | *Phyllomedusa bicolor* | *L. m* | 7.4 | 2 | Lozano et al. 2014 |
| **Bombinin** | *Bombina maxima* | *L. p* | 10.3 | 1.9 | Lozano et al. 2014 |
| **Temporin-SHe** | *Pelophylax saharicus* | *L. b* | 10.5 | 1.6 | André et al. 2020 |
| **Temporin-SHe** | *Pelophylax saharicus* | *L. m* | 11.6 | 1.5 | André et al. 2020 |

* SI: highest value reported when tested on more than one cell type. *L. a - L. amazonensis. L. b - L. braziliensis. L. d - L. donovani. L. i - L. infantum. L. m - L. major. L. p - L. panamensis. L. t - L. tropica.*

**Table S3 – IC₅₀ values and Selectivity Indices (SI) of bioengineered peptides exhibiting leishmanicidal activity against amastigotes.**

| **Peptide** | **Species of origin** | **Species** | **IC50 (μM)** | **SI *** | **Author, year** |
| --- | --- | --- | --- | --- | --- |
| **Mammals** |  |  |  |  |  |
| **RP1** | *Homo sapiens* | *L. a* | 1.3 | 80 | Costa et al., 2020 |
| **Fc-RP1** | *Homo sapiens* | *L. a* | 0.3 | 69 | Costa et al., 2020 |
| **Amphibians/Reptiles** |  |  |  |  |  |
| **Cry3Aa-DS1** | *Phyllomedusa sauvagii* (DS1) | *L. d* | 0.3 | 182.3 | Yang et. al 2019 |
| **Temp-SHd** | *Pelophylax saharicus* | *L. i* | 6.7 | 88.7 | Abbassi et al. 2013 |
| **Cry3Aa-DS1** | *Phyllomedusa sauvagii* (DS1) | *L. a* | 0.7 | 81.6 | Yang et. al 2019 |
| **Temporin [K3]SHa** | *Rana temporaria* | *L. i* | 5 | 71.6 | Raja et al. 2017 |
| **Temporin-SHa** | *Rana temporaria* | *L. i* | 9 | 62.2 | Raja et al. 2017 |
| **Temporin-SHa** | *Rana temporaria* | *L. i* | 20 | 28 | Raja et al. 2017 |
| **Temp-SHd** | *Pelophylax saharicus* | *L. i* | 23.5 | 25.3 | Abbassi et al. 2013 |
| **Temporin [K3]SHa** | *Rana temporaria* | *L. i* | 20 | 17.9 | Raja et al. 2017 |
| **Microorganisms** |  |  |  |  |  |
| **Cry3Aa-DS1** | *Bacillus thuringiensis* (Cry ) | *L. d* | 0.3 | 182.3 | Yang et. al 2019 |
| **Cry3Aa-DS1** | *Bacillus thuringiensis* (Cry ) | *L. a* | 0.7 | 81.6 | Yang et. al 2019 |

* SI: highest value reported when tested on more than one cell type. *L. a - L. amazonensis. L. b - L. braziliensis. L. d - L. donovani. L. i - L. infantum. L. m - L. major. L. p - L. panamensis. L. t - L. tropica.*

**Table S4 – IC₅₀ and Selectivity Indices (SI > 1) of purely synthetic peptides active Against *Leishmania* amastigotes.**

| **Peptide** | **Species** | **IC50 (μM)** | **SI *** | **Author, year** |
| --- | --- | --- | --- | --- |
| **GVL1-TSHa** | *L. a* | 0.3 | 6060.0 | Costa et al., 2023 |
| **GVL1-TSHa** | *L. me* | 0.8 | 2410.0 | Costa et al., 2023 |
| **(GVL1)-pBt** | *L. a* | 0.9 | 2222.0 | Costa et al., 2023 |
| **(GVL1)2-p-Bt** | *L. a* | 2.3 | 889.0 | Costa et al., 2023 |
| **TSHa** | *L. a* | 6.8 | 294.0 | Costa et al., 2023 |
| **p-Bt** | *L. a* | 7.5 | 266.6 | Costa et al., 2023 |
| **TSHa** | *L. me* | 6.7 | 148.0 | Costa et al., 2023 |
| **Syn-safencin 82** | *L. d* | 1.0 | 19.6 | Corman et al., 2022 |
| **Syn-safencin 77** | *L. d* | 1.6 | 12.6 | Corman et al., 2022 |
| **Syn-safencin 7** | *L. d* | 2.0 | 9.8 | Corman et al., 2022 |
| **cecropin A-melittin**  **hybrid peptide** | *L. p* | 4.3 | 8.9 | Fernandes-Reyes et al., 2010 |
| **Syn-safencin 6** | *L. d* | 3.0 | 6.7 | Corman et al., 2022 |
| **Syn-enterocin 73** | *L. d* | 3.4 | 6.0 | Corman et al., 2022 |
| **Syn-enterocin 15** | *L. d* | 3.5 | 5.7 | Corman et al., 2022 |
| **Syn-enterocin 47** | *L. d* | 3.4 | 5.7 | Corman et al., 2022 |
| **Syn-safencin 78** | *L. d* | 3.9 | 5.1 | Corman et al., 2022 |
| **Syn-enterocin 39** | *L. d* | 4.0 | 5.0 | Corman et al., 2022 |
| **CM11** | *L. m* | 9.0 | 4.9 | Khalili et al. 2019 |
| **CM11** | *L. m* | 9.6 | 3.8 | Khalili et al., 2018 |
| **BufIIb** | *L. m* | 25.3 | 1.4 | Esmaeilifallah et al., 2023 |

* SI: highest value reported when tested on more than one cell type. *L. a - L. amazonensis. L. d - L. donovani. L. i - L. infantum. L. m - L. major. L. me - L. mexicana. L. p. - L. pifanoi.*

**Table S5 – IC₅₀ values and Selectivity Indices (SI) of purely synthetic peptides exhibiting leishmanicidal activity against axenic amastigotes.**

| **Peptide** | **Species** | **IC50** | **SI *** | **Author, year** |
| --- | --- | --- | --- | --- |
| **Syn-enterocin 39** | *L. d* | 0.3 | 74.1 | Corman et al., 2022 |
| **Syn-enterocin 47** | *L. d* | 0.3 | 71.7 | Corman et al., 2022 |
| **Syn-enterocin 73** | *L. d* | 0.4 | 56.5 | Corman et al., 2022 |
| **Syn-enterocin 15** | *L. d* | 0.4 | 55.7 | Corman et al., 2022 |
| **Syn-safencin 82** | *L. d* | 375.0 | 53.3 | Corman et al., 2022 |
| **Syn-safencin 77** | *L. d* | 0.4 | 51.3 | Corman et al., 2022 |
| **Syn-safencin 6** | *L. d* | 0.5 | 41.7 | Corman et al., 2022 |
| **Syn-larvacin 35** | *L. d* | 0.6 | 32.0 | Corman et al., 2022 |
| **Syn-safencin 7** | *L. d* | 0.8 | 25.9 | Corman et al., 2022 |
| **Syn-safencin 78** | *L. d* | 0.9 | 21.2 | Corman et al., 2022 |

* SI: highest value reported when tested on more than one cell type. *L. d - L. donovani.*

**Table S6 – IC₅₀ and Selectivity Indices (SI > 1) of purely synthetic peptides active Against *Leishmania* promastigotes.**

| **Peptide** | **Species** | **IC50** | **SI *** | **Author, year** |
| --- | --- | --- | --- | --- |
| **cecropin A-melittin**  **hybrid peptide** | *L. d* | 1.8 | 26.8 | Fernandes-Reyes et al., 2010 |
| **CM11** | *L. m* | 6.9 | 6.4 | Khalili et al. 2019 |
| **BufIIb** | *L. m* | 30.4 | 1.2 | Esmaeilifallah et al., 2023 |
| **CM11** | *L. m* | 7.0 | 1.1 | Esmaeilifallah et al., 2023 |

* SI: highest value reported when tested on more than one cell type. *L. a - L. amazonensis. L. d - L. donovani. L. i - L. infantum. L. m - L. major.*

**Table S7 – IC₅₀ values and Selectivity Indices (SI) of reptile- or amphibian-derived peptides exhibiting leishmanicidal activity.**

| **Peptide** | **Species of origin** | **Species** | **Form** | **IC50 (μM)** | **SI *** | **Author, year** |
| --- | --- | --- | --- | --- | --- | --- |
| **Phylloseptin 7** | *Phyllomedusa nordestina* | *L. i* | promastigote | 10.1 | 3.4 | Pinto et al., 2013 |
| **Temporin-1Sa** | *Pelophylax saharica* | *L. i* | promastigote | 18.1 | 1.4 | Abasi et al., 2008 |
| **Temporin-1Sa** | *Pelophylax saharica* | *L. i* | amastigote | 22.8 | 1.1 | Abasi et al., 2008 |

* SI: highest value reported when tested on more than one cell type. *L. i - L. infantum*

**Table S9 – IC₅₀ values and Selectivity Indices (SI) of microorganism-derived peptides exhibiting leishmanicidal activity.**

| **Peptide** | **Species of origin** | **Species** | **Form** | **IC50 (μM)** | **SI *** | **Author, year** |
| --- | --- | --- | --- | --- | --- | --- |
| antiamoebin (AAM) | *Emericellopsis synnematicola* | *L. i* | amastigote | 7.5 | 5.4 | Fragiadaki et. al, 2018 |
| suzukacillin (SZ) | *Trichoderma viride* | *L. i* | amastigote | 7.6 | 3.0 | Fragiadaki et. al, 2018 |
| antiamoebin (AAM) | *Emericellopsis synnematicola* | *L. i* | promastigote | 8.7 | 4.6 | Fragiadaki et. al, 2018 |
| suzukacillin (SZ) | *Trichoderma viride* | *L. i* | promastigote | 8.2 | 2.7 | Fragiadaki et. al, 2018 |

* SI: highest value reported when tested on more than one cell type. *L. i - L. infantum*
